# Correspondence

**Published:** 1886-08-20

**Authors:** P. Fitz-James


### CORRESPONDENCE.

*Drs. Powell and Word, Editors Southern Medical Record.*

GENTLEMEN: I have been wanting to do something that would show how one who is out of the current looks upon those who are carried along by it to fame and distinction in the medical profession. Perhaps my position may be compared by some one to that of the individual who waited on the brink of the pool for some kind person to come and put him in when the waters were moved. I don't have much inclination to be mixed up thus with a crowd of common people, and unless you could give me a professorship in the Southern Medical College, my aim would be to get elected to a place in the "Association of American Physicians," recently organized, with the express stipulation that it should be limited to one hundred members. I find that there are a few vacancies yet to be filled, and as they are only to be given to "Representative men of the profession," it strikes me as the very best thing for my reputation to get in with some of my old friends, who have elected themselves to constitute this select body. Now, you see, there may be others who are otherwise known by their works in the different practical departments; but being one of a hundred representative men, will place me in the front rank of the profession, not only in the estimation of American practitioners, but throughout the world; and this is a distinction of no small importance. I think after this position is secured your Southern Medical College would feel itself honored by having my name associated with it, and if there is no other place open for me, I would accept a position as emeritus professor in any branch you may think proper to give me. In the meantime, I would be glad if you would enlist the new Governor as well as the old one, in my behalf, and get letters of recommendation from your Congressmen and Senators to some of those prominent physicians who have founded this great Association of American Physicians, recommending me as a suitable person to be elected by them for the future advancement of medical science. Most respectfully yours,

P. FITZ-JAMES, M.D.

